# Conversion of Lignin to Nitrogenous Chemicals and Functional Materials

**DOI:** 10.3390/ma17205110

**Published:** 2024-10-19

**Authors:** Yan Li, Jingrong Li, Bo Ren, Haiyang Cheng

**Affiliations:** 1 Jilin Provincial Key Laboratory of Straw-Based Functional Materials, Institute for Interdisciplinary Biomass Functional Materials Studies, Jilin Engineering Normal University, Changchun 130052, China; yanlicc0206@163.com (Y.L.); renbo100@126.com (B.R.); 2 Jilin Province Key Laboratory of Green Chemistry and Process, Changchun Institute of Applied Chemistry, Chinese Academy of Sciences, Changchun 130022, China; lijr@ciac.ac.cn

**Keywords:** biomass, lignin, modifications, nitrogenous functional materials, applications, wastewater treatment

## Abstract

Lignin has long been regarded as waste, readily separated and discarded from the pulp and paper industry. However, as the most abundant aromatic renewable biopolymer in nature, lignin can replace petroleum resources to prepare chemicals containing benzene rings. Therefore, the high-value transformation of lignin has attracted the interest of both academia and industry. Nitrogen-containing compounds and functionalized materials are a class of compounds that have wide applications in chemistry, materials science, energy storage, and other fields. Converting lignin into nitrogenous chemicals and materials is a high-value utilization pathway. Currently, there is a large amount of literature exploring the conversion of lignin. However, a comprehensive review of the transformation of lignin to nitrogenous compounds is lacking. The research progress of lignin conversion to nitrogenous chemicals and functional materials is reviewed in this article. This article provides an overview of the chemical structure and types of industrial lignin, methods of lignin modification, as well as nitrogen-containing chemicals and functional materials prepared from various types of lignin, including their applications in wastewater treatment, slow-release fertilizer, adhesive, coating, and biomedical fields. In addition, the challenges and limitations of nitrogenous lignin-based materials encountered during the development of applications are also discussed. It is believed that this review will act as a key reference and inspiration for researchers in the biomass and material field.

## 1. Introduction

With the advancement of technology and the improvement of environmental protection awareness, biomass has been extensively utilized and developed and is considered to be the best substitute for fossil resources. According to the Statistical Review of World Energy, fossil fuels such as coal, oil, and gas accounted for up to 81% of global primary energy consumption in 2023. Lignocellulosic biomass is the most abundant and important renewable resource on Earth, produced from plant materials such as wood, hemp plants, crop straw, bagasse, corncob, sawdust, rice husk, soybean husk, and cassava residue [1]. Lignin is a complex aromatic natural polymer that is present in lignocellulosic biomass (15–30% by weight), together with cellulose, hemicelluloses, and other trace substances. Approximately 150 billion tons of natural lignin are derived from plant growth each year [2]. Currently, technical lignin is mostly produced as byproducts in the process of paper making, ethanol production from lignocellulosic, and other biomass refining industries. In the pulp and paper industry alone, the estimated production capacity by 2025 is approximately 134.5 million tons of chemical pulping (mainly Kraft pulping), and the production of lignin as a byproduct can range from 67 to 81 million tons [3]. Most lignin (95%) is burned as a low-cost fuel source for generating heat or electricity. This not only wastes a lot of resources but also causes environmental pollution. Therefore, the high-value utilization of lignin has received widespread attention from researchers [4,5,6,7,8,9,10,11,12,13,14,15,16].

Heteroatom nitrogen refers to the introduction of nitrogen as a non-carbon atom into the molecular structures of organic or inorganic compounds. Nitrogen atoms play an important role in many chemical reactions and materials due to their high electronegativity and ability to form multiple chemical bonds. Hence, nitrogenous chemicals and materials are essential in many aspects of modern life and widely used in advanced functional materials, pharmaceuticals, pesticides, nutrition products, textiles, polymers, and energy storage. However, currently, most nitrogenous chemicals and materials are produced by fossil fuel refineries through chemical reactions with ammonia or ammonia-derived feedstocks [17]. Lignin, as the only aromatic renewable natural polymer with a three-dimensional network structure, can be converted into nitrogenous chemicals and functional materials, reducing dependence on non-renewable resources while avoiding waste of lignin and realizing the value-added lignin resources.

In recent years, only limited reviews have been published on the production of lignin-based nitrogen-containing chemicals and functionalization materials, with more attention paid to lignin-derived chemicals as the raw materials, catalyst design, and reaction mechanisms of catalytic amination. For example, Rong et al. [18] reviewed the conversion of lignin-derived phenols, ethers, carbonyl compounds, and alcohols into nitrogen-containing compounds such as aniline and benzylamine through heterogeneous catalytic amination, with a focus on cheap catalysts, green solvent systems, nitrogen source systems, and mild reaction conditions. Jayaramudu et al. [19] reviewed the Mannich reaction of lignin with different types of amines and discussed the applications of Mannich-modified lignin. The Mannich reaction of lignin is one of the most successful and popular modification methods used to prepare lignin-based nitrogen compounds, which can modify and add active amine sites in the lignin material. In addition, lignin can be partially non-renewable petroleum-derived polyols in the manufacturing of PU, which is one of the most widely used polymer materials in the industry. Recently, there have been several reviews on lignin-based lignin polyurethane (PU) [20,21,22]. Moreover, using lignin as a carbon source to prepare carbon–nitrogen materials is also a kind of lignin-based nitrogenous material that can be widely used in the catalytic field. Hence, to promote the high value of lignin utilization, a state-of-the-art review is conducted for the latest progress in the synthesis of nitrogenous chemicals and functionalization materials using lignin as the starting molecule. Firstly, the basic structure and properties of lignin, as well as the types of technical lignin, are introduced. Secondly, the preparations of various types of nitrogenous lignin-based material are summarized, including modified lignin with nitrogen-containing functional groups, lignin-based polyurethane, and nitrogenous lignin-based carbon materials. Thirdly, the applications of nitrogenous functionalized lignin materials in wastewater treatment, slow-release fertilizer, adhesive, coating, and biomedical fields are reviewed. Finally, insights and perspectives into the further development of lignin-derived nitrogenous functionalization materials are proposed.

## 2. Lignin

### 2.1. Structure and Properties of Lignin

The original form of lignin is typically a white or nearly colorless aggregate, constituting 15–35% of the dry weight in most plants, depending on their origin and species. In lignocellulosic biomass, lignin accounts for 27–33% of softwood, 18–25% of hardwood, and 17–24% of grass [23]. Due to differences in raw material sources and separation methods, the properties of the separated lignin, such as the color, molecular weight, and solubility, can vary significantly from those of the original lignin.

**Figure 1:**
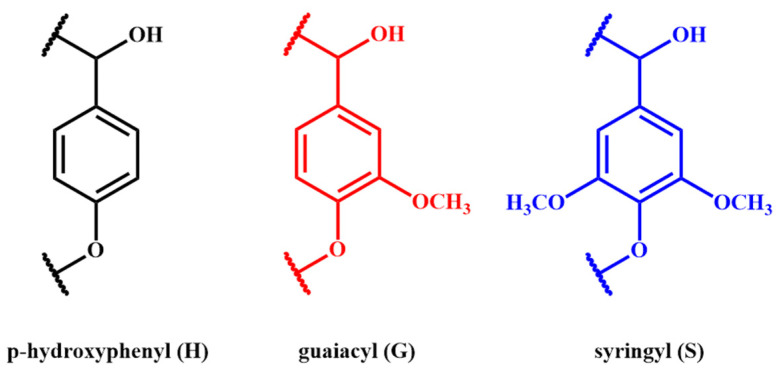
Three main phenolic structures present in lignin.

Lignin is a non-crystalline and amorphous phenolic polymer with a three-dimensional network structure that is random and quite complex [24]. So far, studies on lignin have not yet clarified its precise chemical structure. In general, lignin is mainly composed of three phenolic structures: p-hydroxyphenyl (4-hydroxyphenyl, H), guaiacyl (4-hydroxy-3-methoxyphenyl, G), and syringyl (4-hydroxy-3, 5-dimethoxyphenyl, S) (Figure 1) [25]. Lignin polymerization starts from the transformation of these three monomers into their radical form by peroxidases and laccases. Then, they react together in a combinatorial way by free-coupling mechanisms without any enzymatic control. Consequently, contrary to cellulose and other polymers, lignin is arranged in a hyperbranched topology with no regular repeating structure. But, overall, the structure and three monomers of lignin are closely influenced by a series of factors such as plant species, the growth environment, and growth duration [26]. For example, softwood contains 25–30% G monomers, hardwood contains 16–24% G and S monomers, and grasses contain 13–18% G, S, and H monomers [27].

**Figure 2:**
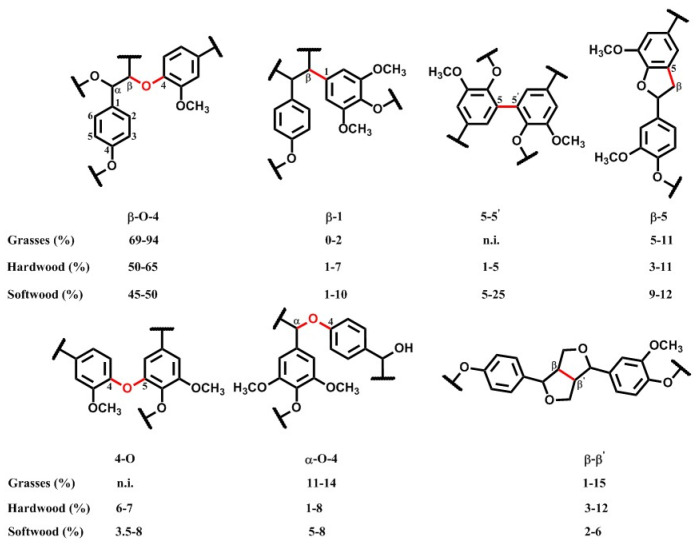
Typical bonds of lignin.

Different types of ether bonds and carbon–carbon bonds are generated during the combination of radicals, such as β-O-4 (phenylpropane β-aryl ether), α-O-4 (phenylpropane α-aryl ether), 4-O-5 (diaryl ether), β-1 (1,2-diaryl propane), β-β (pinoresinol), 5-5′ (biphenyl), and β-5 (phenylcoumaran) (Figure 2) [3]. Among them, the β-O-4 ether bond is the most abundant linkage type. In addition, there are various functional groups such as phenolic hydroxyl, alcohol hydroxyl, methoxy, and aldehyde groups in the lignin structure. These structural characteristics enable lignin to be transformed into high-value chemicals and functional materials through a series of chemical modification processes.

### 2.2. Types of Technical Lignin

Technical lignin is extracted from biomass by different methods. The composition and characteristics of each technical lignin will vary depending on its source and extraction method (Table 1) [28,29,30,31,32].

#### 2.2.1. Alkali Lignin

Alkali lignin comes from the alkaline chemical pulping process. Alkali lignin is mainly Kraft lignin, which is the lignin separated from the kraft pulping process. Kraft lignin is a byproduct of the Kraft process, which is a widely used method for pulping and paper production. During the Kraft process, the sawdust is treated with a mixture solution of sodium hydroxide and sodium sulfide at high temperatures (150–170 °C) and high pressure (3~5.5 kg/cm^2^) for several hours. After Kraft treatment, nearly 90% of lignin in wood dissolves together with other organic substances in the cooking liquor to form black liquor. The solid precipitate of Kraft lignin can be obtained by acidifying the black liquor to a pH value of 5.0 or lower by adding acid. Kraft lignin has a high degree of polymerization and cross-linking, which makes it relatively resistant to degradation and chemical modification. Meanwhile, the solubility of Kraft lignin in water and most organic solvents is low, which limits its processing ability and reactivity. Hence, the main application of Kraft lignin at present is as a fuel for energy production.

#### 2.2.2. Lignosulfonates

Lignosulfonates are generated during the sulfite pulping process of wood, and they are water-soluble and anionic due to the presence of sulfonic groups attached to the benzylic carbon atoms in their structures. In addition, lignosulfonates are also soluble in some organic solvents, such as ethylene glycol, propylene glycol, and dimethyl sulfoxide. After sulfite pulping, lignosulfonates are filtered out from the pulp as waste liquid. The waste liquid contains about 50–80 wt.% lignocellulose, 30 wt.% hemicellulose, and 10 wt.% inorganic matter. The average Mw of lignosulfonates extracted from hardwood and softwood is between 6000 and 12,000 Da.

#### 2.2.3. Organosolv Lignin

Organosolv lignin is obtained by using organic solvents such as methanol, ethanol, acetone, and ethylene glycol, and catalysts are usually added to improve the pulping rates. Compared with other lignin extraction methods shown in Table 1, organosolv lignin is considered to have the characteristics of high purity, low ash, a low carbohydrate content, a low molecular weight, and being sulfur-free. Furthermore, the reactivity of organosolv lignin is affected by the preservation of internal β-O-4 linkages, making it suitable for chemical transformations. Therefore, organosolv lignin is an attractive feedstock for various applications, such as bioplastics, biofuels, chemicals, and other high-value-added products.

#### 2.2.4. Enzymatic Lignin

Enzymatic lignin is a residue that cannot react with enzymes in the production of bioethanol from lignocellulose with various enzymes. Enzymatic lignin contains 50–75% lignin and untreated cellulose, oligosaccharides, nitrogen-containing compounds, etc. Due to the low purity and solubility of enzyme lignin, its application is limited. Thus, the main application of enzymatic lignin is similar to that of Kraft lignin, which is burned as fuel and generates heat for energy production.

## 3. Preparation of Nitrogenous Functionalized Lignin Materials

### 3.1. Modified Methods to Prepared Lignin with Nitrogen-Containing Functional Groups

#### 3.1.1. Ammoxidation

**Figure 3:**
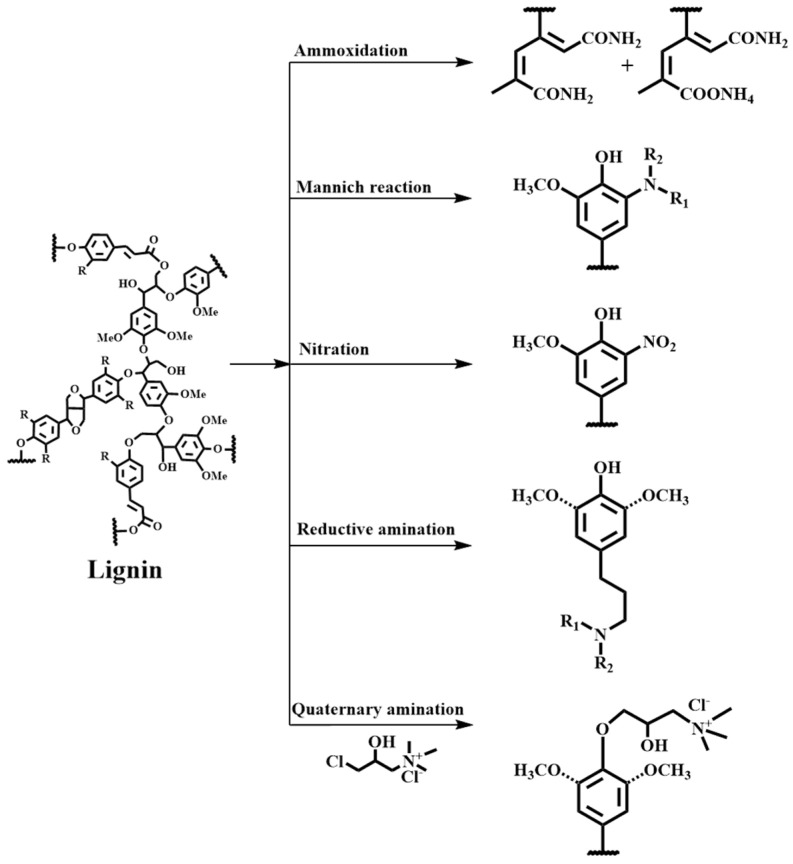
Chemical modified lignin with nitrogen-containing functional groups.

The ammoxidation of lignin is a process involving the reaction of lignin with ammonia under specific pressure and temperature using different oxidants (O_2_, H_2_O_2_, etc.) to produce nitrogen-modified lignin [2]. Figure 3 shows one of the possible pathways in the ammoxidation of lignin. Meier et al. [33] used oxygen as an oxidant in ammonia solution to perform the ammoxidation of five types of lignin (Kraft, organosolv, soda, alkaline-sulphite-anthraquinone-methanol pulping, and fermented sodium lignosulfonate). The order of nitrogen content is Kraft lignin, organosolv lignin, and then lignosulfonate. Under optimal conditions, the maximum nitrogen content is 13–14% lignin. Oxygen pressure, reaction time, and temperature are key parameters in the ammoxidation of lignin, affecting the nitrogen content and C/N ratio in the treated lignin. Qi et al. [34] used hydrogen peroxide as an oxidant to ammoxidize straw-pulp alkaline lignin. The ammoxidized product reached 11.6%, and the ammonia nitrogen content was 4.1%. Cao et al. [35] compared the chemical structures of the three lignosulfonates before and after ammoxidation. The molecular weights of the products decreased, the aromatic ring structures partially degraded, and the nitrogen contents increased, but the C/N ratios decreased. Meanwhile, nitrogen mainly existed in the form of amine, amide, and ring-bonded nitrogen.

#### 3.1.2. Mannich Reaction

The Mannich reaction of lignin is a reaction in which lignin with active hydrogen is simultaneously condensed with formaldehyde and amine, and the active hydrogen is replaced by a substituted or unsubstituted aminomethyl group [36]. In the structural unit of lignin, there are many active hydrogen atoms at the α position of the phenolic hydroxyl group and the α position of the carbonyl group; when amine compounds condense with aldehydes and/or compounds with active hydrogen atoms, the active hydrogen atoms are replaced by aminomethyl groups (Figure 3) [19]. In recent years, the Mannich reaction has been one of the important and most commonly used methods for introducing amino groups into lignin, widely utilized in lignin modification and the preparation of lignin-based functional materials [37]. The Mannich reaction can be carried out under acidic, neutral, or alkaline conditions. Among them, aminated lignin is usually prepared from formaldehyde and various types of amines under alkaline conditions [2]. However, the low chemical reactivity of lignin greatly limits the efficiency of chemical reactions. For example, the Mannich reaction of S-lignin is limited due to the high degree of methoxylation of S-lignin and the low availability of free C5 [38]. Therefore, to obtain more reactive sites, lignin was pretreated by phenolization and mild depolymerization. For instance, lignin could be bonded with phenol via phenolization, and the reactive sites of lignin could be significantly increased. The amination of industrial softwood Kraft lignin was conducted using the Mannich reaction by Du et al. The nitrogen content of aminated lignin was increased from 2.5% to 4.8% by phenolation pretreatment [39].

#### 3.1.3. Nitration

The nitration of lignin usually uses a non-aqueous solvent containing nitric acid to introduce NO or NO_2_ functional groups onto the aromatic ring of lignin. The most widely used nitration agent is a mixture of nitric acid and concentrated acetic acid, acetic anhydride, or sulfuric acid [40]. The obtained nitro-lignin is an amorphous powder with a color varying from yellow to brown, an average molecular weight between 600 and 2000 Da, and a nitrogen doping amount of typically about 7% by weight [41]. Bunton et al. [42] proposed a possible mechanism for the nitration of lignin with nitric acid. Lignin nitration has two steps. The first step was slow nitration to form a quinonoid oxonium ion, and the second step is the removal of a methyl group. During the nitration process, lignin molecules also underwent oxidation, demethoxylation, and degradation reactions. The degree of nitration and the solubility of lignin depended on the nitration conditions and not the particle size [43]. Ahmad et al. successfully carried out the nitration of Kraft lignin in softwood and hardwood using nitric acid under ambient conditions (room temperature and pressure) [44]. The nitrogen content of the obtained nitro-lignin was 5%, and the average molecular weight was 1350 Da, which was much lower than that of the original lignin. Khabarov et al. [45] investigated the nitration of sulfate lignin with nitric acid in a dioxane aqueous solution under homogeneous conditions, resulting in a high nitration degree at 50 °C and a low nitric acid dosage. They proposed that the obtained nitro-lignin may contain one nitro group for every 1.5–2 phenyl-propane units.

#### 3.1.4. Reductive Amination

Reductive amination is achieved by reacting ketones and aldehydes with amines to form carbon–nitrogen double bonds, which are then reduced by a reducing agent. Li et al. [46] reported the first example of lignin synthesizing benzylamines through a two-step method. Firstly, the lignin oil was obtained through the mild depolymerization of lignin catalyzed by a binuclear rhodium complex at 110 °C, resulting in a yield of 8.3 wt.% of aromatic keto-monomers and oligomers. Secondly, they converted lignin oil with pyrrolidine (amine source and reductant) over Pd/C, obtaining a corresponding benzylamine yield of 0.4% by weight. The low yield of benzylamines may be due to the inability of a large number of oligomers to undergo an amination reaction and the competitive hydrogenation reaction of the keto-monomers. Yan et al. [47] reported a single-step catalytic strategy for the direct production of phenolic amines from poplar lignin through reductive fractionation in an aqueous ammonia–alcohol mixture over Ru catalyst in the presence of H_2_ (Figure 3). According to the type of alcohol used, primary, secondary, and tertiary phenolic amines can be synthesized. The maximum yield of phenolic amines was 26.6% based on lignin content. During processes including delignification, amination, and alkylation, native lignin was selectively removed from woody biomass, leaving behind cellulose, hemicellulose, and other constituents that were largely unaffected. Integrating the reductive amination reaction into the lignin reduction and fractionation process to produce valuable organonitrogen chemicals in the lignin treatment step may be a significant advancement in the lignin-first strategy [48].

#### 3.1.5. Quaternary Amination

In the quaternary amination modification of lignin, the phenolic hydroxyl groups or other active sites in lignin molecules react with quaternization reagents through chemical modification methods such as graft copolymerization or substitution reaction to form positively charged quaternized lignin, endowing lignin with cationic properties. Yang et al. [49] synthesized quaternized lignin using 2,3-epoxypropyldim-ethylallyl ammonium chloride as a quaternary ammonium salt monomer through grafting copolymerization under a nitrogen atmosphere. Zheng et al. [50] prepared quaternized lignosulfonate (QL) by substitution reaction with 3-chloro-2-hydroxypropyl trimethylammonium chloride as a quaternary ammonium reagent (Figure 3); the nitrogen content in QL was 8.53 wt.%. In contrast to the monomers with only one active site attached to the lignin, Saini et al. [51] first polymerized a bifunctional molecule poly (vinylbenzyl chloride) onto lignin to generate benzyl chloride groups, which were then quaternized with 1-methylimidazole or 1-n-butylimidazole to form cationic vinyl-imidazole lignin; the nitrogen contents were 11.42% and 9.00%, respectively.

### 3.2. Lignin-Based Polyurethanes

Polyurethanes (PUs) are a family of versatile polymers formed by the polyaddition reaction between a polyol and a di- or tri-isocyanate. They are renowned for their durability, flexibility, resilience, and excellent insulation properties [52,53,54]. Lignin is usually employed as a green alternative to conventional polyols due to its high number of hydroxyl groups, aromatic structure, and easy availability. This has greatly aroused interest in both academia and industry [55,56,57,58,59]. Depending on its interaction with isocyanate, lignin can act either as a filler or as a reagent. For example, Bello and Yan used lignin-containing nanocellulose fibrils (LCNFs) as mechanical reinforcements for rigid PU foams (RPUFs) with the goal of maintaining the mechanical performance of the foam while using less isocyanate. When the optimal addition amount is 0.1 wt.%, LCNF-reinforced foam with lower isocyanate content (a 4.5% reduction) showed fairly standardized compressive strength and stiffness compared with foam with a higher isocyanate content, which proves their ability to compensate for the reduction in hard segment when the isocyanate content is lower [60]. However, the direct use of lignin as the only polyol is limited by its low reactivity with isocyanate groups, resulting in products with suboptimal properties. Therefore, lignin usually is used in PU production in two ways: (1) directly using lignin to prepare polyurethane and (2) using modified lignin to prepare polyurethane.

#### 3.2.1. Directly Using Lignin to Prepare Polyurethanes

Since the unmodified lignin has high polarity and the polyol has relatively low polarity, the compatibility of these two materials is poor. Therefore, to minimize the poor miscibility of unmodified lignin in traditional petroleum-based polyols, the addition of unmodified lignin as a partial substitute for petroleum-based polyols is usually limited to less than 30 wt.% to achieve acceptable performance in the PU. Haridevan et al. [61] used diols (ethylene oxide diols, propylene oxide diols) and glycerol as compatibilizers and cross-linkers to improve the dispersion of Kraft lignin in polyols, and explored the dispersion and solubility of Kraft lignin in different types of polyols at room temperature to produce designed polyurethane materials, using microscopic, gravimetric, and rheological analyses. In addition, the performance of PU can be improved by heating the polyol/lignin dispersions at 120 °C before the reaction, which enhances the disaggregation of lignin microparticles, resulting in better lignin dispersion in the polyol system [62]. Alternatively, the molecular weight of Kraft lignin can be reduced by solvent fractionation, such as acetone–methanol [63] and ionic liquids [64]. This approach can also improve the miscibility and dispersion of Kraft lignin in polyols and consequently enhance the properties of the resulting PU. However, commercializing low-molecular-weight lignin produced by solvent fractionation has been economically unfeasible to date [65].

#### 3.2.2. Using Modified Lignin to Prepare Polyurethane

Due to the complex structure of lignin, the resulting steric effect greatly reduced the number of available reaction sites, so only a small portion of the hydroxyl group could cross-link with the isocyanates. Moreover, only partial lignin can be incorporated into the polymer, since lignin possesses intrinsic brittleness and low solubility. To ameliorate these problems, lignin modification is considered to be one of the most effective approaches, and it includes fractionating lignin to oligomeric feedstock or modifying lignin to increase new chemical active sites. Liu et al. [66] successfully synthesized high-performance polyurethane from depolymerized enzymatic hydrolysis lignin, polytetramethylene ether glycol, and hexamethylene diisocyanate. Smit et al. [67] produced polyurethane with high lignin content and tunable properties from catalytically reduced beech wood lignin and hexamethylene diisocyanate. The chemical modification of lignin has been widely studied to increase the content of the hydroxyl group and improve the reactivity, as well as to enable the creation of modified lignin more suitable for producing PU, such as hydrox alkylation, oxy alkylation, and dealkylation. Wang et al. [68] synthesized PEG-2000 grafted alkali lignin (AL-PEG2000), which was subsequently employed as a partial substitute for petroleum-derived polyols to prepare highly resilient PU foams. Chen et al. [69] prepared PU adhesives with high thermal stability and mechanical properties from demethylated lignin, in which a 36.5% increase in hydroxyl content was obtained under optimal conditions. Vieira et al. [70] optimized the oxy alkylation of Kraft lignin with propylene carbonate (PC) to produce different PU products, such as rigid foams and adhesives.

**Figure 4:**
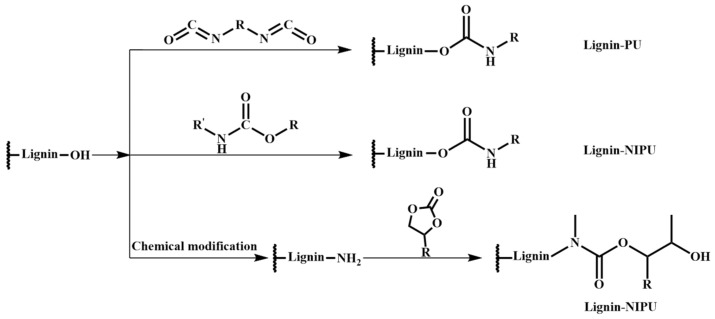
Chemical routes used to produce lignin-based PU and NIPU.

The presence of toxic insufficiently reacted isocyanate in PU synthesis affects the safety of its subsequent use. In this context, non-isocyanate-based polyurethanes (NIPUs), which do not use toxic isocyanates as raw materials, have received much attention as potential alternatives to traditional polyurethanes (Figure 4) [71,72,73,74,75,76]. Sternberg and Pilla [77] produced NIPU from cyclocarbonated Kraft lignin and dimer diamine. This approach benefited from the high reactivity between cyclocarbonates and diamines, leading to the rapid gelation of the reaction mixture. The cyclocarbonated Kraft lignin was synthesized by the oxy alkylation of Kraft lignin with glycerol carbonate, followed by cyclocarbonation with dimethyl carbonate in the presence of K_2_CO_3_. Arias et al. [78] prepared NIPU adhesives for wood panels by reacting Kraft lignin and organosolv lignin with dimethyl carbonate, followed by a reaction with hexamethylenediamine. Meng et al. [79] produced NIPU from aminated lignin (via the Manich reaction) and bicyclic carbonates. This novel bio-based NIPU showed good thermal stability and high tensile strength.

### 3.3. Nitrogenous Lignin-Based Carbon Materials

Nitrogen-doped carbon materials contain active carbon centers near the N atoms, which are beneficial for improving catalytic performance and are widely used in fuel cells, electrocatalysis, supercapacitors, energy storage, and heterogeneous catalysis [80,81,82,83,84,85,86,87,88,89]. Lignin is a good carbon source choice for carbon-based catalysts because it is an amorphous three-dimensional network structure rich in C–C and C–O bonds. During the pyrolysis process, lignin is gradually decomposed and the obtained carbon source is stable and not easy to volatilize. For the application of carbon materials from nitrogenous lignin, Dong et al. [90] introduced the amino group into the lignin structure and then chelated Ni and Co. Finally, N-doped carbon-based metal catalyst was obtained after pyrolysis, which had a good catalytic activity and stability in oxygen reduction and oxygen evolution reactions. The promoting effect is not only achieved by doping nitrogen atoms into the lignin structure to enhance catalytic activity but also by firmly inserting Ni and Co into the carbon skeleton and uniformly dispersing them in the carbon base after carbonization. An et al. [91] synthesize an aminated lignin-derived sponge carbon (LC-PG) for the capacitive removal of Cu^2+^ ions. LC-PG has a large specific surface area of 558.3 m^2^⋅g^−1^ with an interconnected pore structure. LC-PG exhibits excellent capacitive adsorption (53.75 mg⋅g^−1^) of Cu^2+^ ions at 1.2 V. Guo et al. [92] prepared high nitrogen-containing carbon with pyrrolic-N species (Chem-ACM) from lignin for Zn-ion hybrid supercapacitors. Chem-ACM has a high surface area 2D graphene-like architecture, obtained through a Mannich reaction with ethylenediamine and formaldehyde, carbonization, and activation. As a cathode, Chem-ACM achieves a high storage capacity of 161.2 mA⋅h^−1^⋅g^−1^ at 1 A⋅g^−1^, an energy density of 106.7 W⋅h^−1^⋅kg^−1^ at 897 W⋅kg^−1^, and an excellent retention capacity (94%) after 10,000 cycles.

## 4. Applications of Nitrogenous Functionalized Lignin Materials

### 4.1. Application in Wastewater Treatment

With the rapid growth of global industrial production and the continuous increase in resource development, how to control water pollution has become a severe and urgent challenge. Wastewater contains large amounts of organic and inorganic toxic substances, such as heavy metal ions, phenol and its derivatives, organic dyes, inorganic anions, pesticide compounds, antibiotics, and other medical compounds [27,31,93,94,95]. Therefore, there is an urgent need for special materials and technologies for wastewater treatment. Due to the high porosity of the lignin structure and the abundance of active functional groups such as phenolic hydroxyl, hydroxyl, aldehyde, and carbonyl groups in lignin molecules, lignin can directly remove pollutants from water through physical adsorption, ion exchange, and chelation [26,96]. However, the effectiveness of directly removing pollutants from water by lignin alone is limited. Therefore, to improve the removal ability, researchers have modified lignin or prepared other lignin-based materials. For example, amine-functionalized lignin is one of the commonly used materials in wastewater treatment [23,97,98,99]. Table 2 summarizes recent research on the application of amine-modified lignin in wastewater treatment.

Wang et al. prepared lignin-based flocculants by copolymerizing Kraft lignin with acrylamide in a UV-induced aqueous copolymerization system and then modified by a Mannich reaction with formaldehyde and diethylenetriamine. Two different types of lignin-based flocculant (LBF1 and LBF2) could be precisely controlled by adjusting the dosage of formaldehyde and diethylenetriamine. The turbidity removal rate of kaolin suspension by LBF1 could reach 99.3%, and the decolorization rates of Acid Black 1, Direct Red 80, and Reactive Red 2 solutions using LBF2 exceeded 99.6%, being superior to almost all other lignin-based flocculants previously reported [100]. In addition, they used ethyl acetate-soluble hydrothermal lignin and lignosulfonate to copolymerize with (2-methacryloyloxyethyl) trimethyl ammonium chloride under ultraviolet light to synthesize amine-functionalized lignin flocculants. The removal rates of E. coli, kaolin, and Acid Black 1 were 99.6%, 99.2%, and 95%, respectively [101,102].

**Figure 5:**
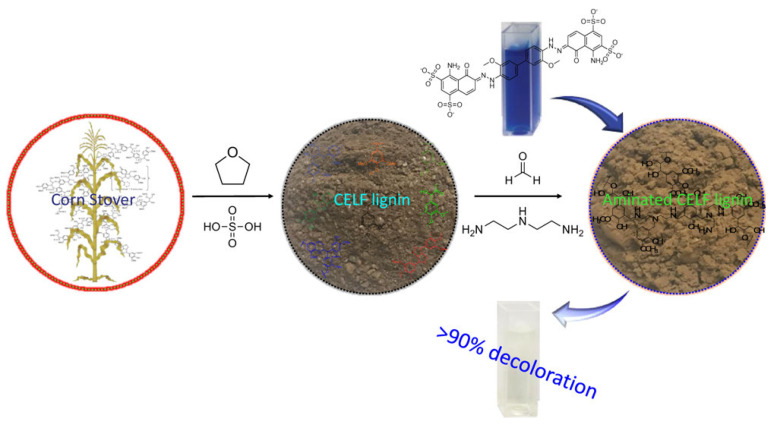
Amine-modified lignin by organosolv lignin to remove DB 1 dye from Ref. [103]. Copyright 2020, The American Chemical Society.

Except for Acid Black 1, Direct Red 80, and Reactive Red 2, many studies have reported the use of amine-modified lignin materials to adsorb other dyes from wastewater (Table 2) [103,104,105,106]. Meng et al. [103] prepared amine-modified lignin by copolymerizing organosolv lignin (THF and water pretreatment corn stover) with diethylenetriamine and formaldehyde to remove DB 1 dye (Figure 5). The removal rate of DB 1 dye exceeded 90%, and the maximum adsorption capacity of DB 1 dye was 502.7 mg⋅g^−1^. To obtain more effective functional groups of lignin, amine-modified lignin was prepared from Kraft lignin through oxidation and amination processes. Compared with the direct amination method, the aminated lignin after oxidization had the highest removal rate of malachite green, reaching 99.1% in [104]. In addition, to promote the separation and recovery of adsorbents from aqueous media, magnetic nanoparticles (e.g., Fe_3_O_4_) were introduced to amine-modified lignin materials. For example, Du et al. [105] prepared lignin-based magnetic nanoparticles by copolymerizing fractionated alkaline lignin with aminopropyl triethoxy silane and formaldehyde and then cross-linking them onto the surface of magnetic Fe_3_O_4_ nanoparticles to remove methylene blue. The adsorption capacity for methylene blue was 234.27 mg⋅g^−1^. Zong et al. [106] prepared magnetically recyclable aminated lignin materials (EL-PEI@Fe_3_O_4_-Mg) for the removal of Congo red. Mg(OH)_2_ had a positively charged surface and strong electrostatic attraction to anionic dyes. The combination of Mg(OH)_2_ and Fe_3_O_4_ particles decorated with aminated lignin can not only improve the adsorption performance of Congo red but also make the adsorbent easy to separate from water. The removal rate of Congo red dye was 92% after five cycles.

Lignin-based adsorbent materials are also widely used for the removal of metal ions from wastewater [23,97,98]. Amine-functionalized lignin materials have shown good performance in adsorbing heavy metal ions (Table 2) [107,108,109,110,111,112]. Guo et al. [107] synthesized amine-functionalized lignin microspheres through an inverse suspension polymerization method and achieved highly ultrafast U(VI) adsorption and desorption through a fixed-bed column system under a high-salinity condition. They not only purified U-contaminated nuclear wastewater but also successfully achieved U(VI) from nuclear wastewater. Xiao et al. [108] reported the creation of a lignin-based nano-trap (LBNT) through aminated alkaline lignin to remove different types of heavy metal ions. The adsorption efficiencies of LBNT for both borderline ions, such as Pb (II), Zn (II), and Cu (II), and soft ions, such as Ag(I), Cd (II), and Hg (I), exceeded 99%. Moreover, the silver-loaded nanocomposites (Ag@LBNT) had high bacteriostatic rates against E. coli (99.7%) and Staphylococcus aureus (99.8%). Similarly, to improve the separation of adsorbents from water, amine-functionalized magnetic lignin nanoparticles were prepared to remove metal ions [109,110].

Excessive phosphate concentration (>10 mg⋅L^−1^) can lead to eutrophication, reduce the quality of the aquatic environment, and even pose a risk to human life and aquatic communities. Low concentrations of phosphate are adsorbed from water or wastewater to prevent eutrophication. Luo et al. [113] chelated Fe(III) onto aminated lignin to remove phosphate. The adsorption removal rate of phosphate exceeded 90%.

### 4.2. Application in Slow-Release Fertilizer

Nitrogen plays an important role in plant growth. The application of nitrogen fertilizer (the amount and form of nitrogen application) not only directly affects yield but also determines quality [114,115]. Therefore, to achieve a high yield and ensure crop quality, it is very important to apply nitrogen fertilizer reasonably in agricultural production. However, about 40–70% of the nitrogen in traditional mineral nitrogen fertilizers (such as urea) is leached from the soil and lost through various channels (transformations and movements), which leads to environmental pollution and ultimately directly and indirectly affects human health [24,116]. Hence, the development of slow-release fertilizers is an effective way to improve nitrogen fertilizer utilization efficiency [117,118]. Lignin and its derivatives have attracted considerable attention as novel eco-friendly fertilizers, as their application have been proven to have positive effects on plant development, soil microorganism dynamics, and ultimate soil fertility [25]. For example, lignin itself can effectively inhibit the activity of urease in soil, allowing urea to stay in the soil for a longer period. Lignin, as a precursor of humic acid, can be completely degraded to humus by soil microorganisms, thereby increasing the content of soil organic matter and improving soil fertility. Moreover, amine-functionalized lignin fertilizers and materials can also be used as slow-release nitrogen fertilizers.

In the early days, the nitrogen content of lignin was increased mainly through the ammoxidation of lignin and then used for the slow release of nitrogen fertilizer [33,119,120,121]. Ramírez-Cano et al. [119] studied the effect of a single application of ammoxidized Kraft lignin or urea during two growth cycles of sorghum. At the end of the first cycle, plants using urea gained greater biomass. However, in the second growth cycle, plants using ammoxidized Kraft lignin performed better. This difference is due to the rapid mineralization and subsequent leaching of nitrogen in urea during the first cycle, while nitrogen in ammoxidized Kraft lignin can be slowly released at a rate sufficient to sustain plant development in the second growth cycle. In recent years, amine-functionalized lignin fertilizers have been prepared through the Mannich reaction to enhance nitrogen content [37,122,123]. To increase the active sites in lignin feedstock, phenolization is employed before the Mannich reaction. Jiao et al. [37] developed a phenol biorefining technique for lignin, and the number of active sites in lignin was significantly increased from an initial 2.91 mmol⋅g^−1^ to 8.26 mmol⋅g^−1^. Subsequently, phenolated lignin was aminated under alkaline conditions through the Mannich reaction with different amines and formaldehyde. The nitrogen content in the aminated lignin highly depended on the type of amination reagent, rather than the proportion of reactants. The highest nitrogen content was 10.1% and the lowest C/N ratio was 6.08 under optimal conditions. Then, they compared the content of ammonium and nitrate ions leached from soils with added aminated lignin or urea. In urea-amended soil, most of the nitrogen was rapidly released within the first 28 days. After 28 days, the leaching amount of these two ions using aminated lignin as a nitrogen source continued to gradually increase, indicating that the mineralization rate of aminated lignin was much slower than that of urea. Recently, Wang et al. successfully prepared amine-functionalized lignin slow-release nitrogen fertilizers through phenolization and amination modification under different microwave heating conditions [122]. The highest organic nitrogen content (7.84%) was obtained under the conditions of 8.0 g ethylenediamine at 80 °C for 40 min. The advantages of microwave heating are its short processing time and simple operation. Subsequently, Wang et al. [123] prepared aminated lignin from phenolized lignin with ethylenediamine and formaldehyde through the Mannich reaction under alkaline conditions. For the prepared aminated lignin, the content of mineral nitrogen and available nitrogen was increased significantly. The first-order kinetic equation of soil nitrogen mineralization indicates that aminated lignin significantly increases the potential for nitrogen mineralization (84.7–143.9%). In addition, aminated lignin was applied for the first time in the remediation of cadmium (Cd) pollution in soil, which could efficiently reduce the effectiveness of Cd through direct (self-adsorption) and indirect effects (improvement of soil pH, soil organic matter, and reduction in soil zeta potential), thus realizing Cd passivation in soil. There is great significance for facilitating the sustainable development of agricultural production.

**Figure 6:**
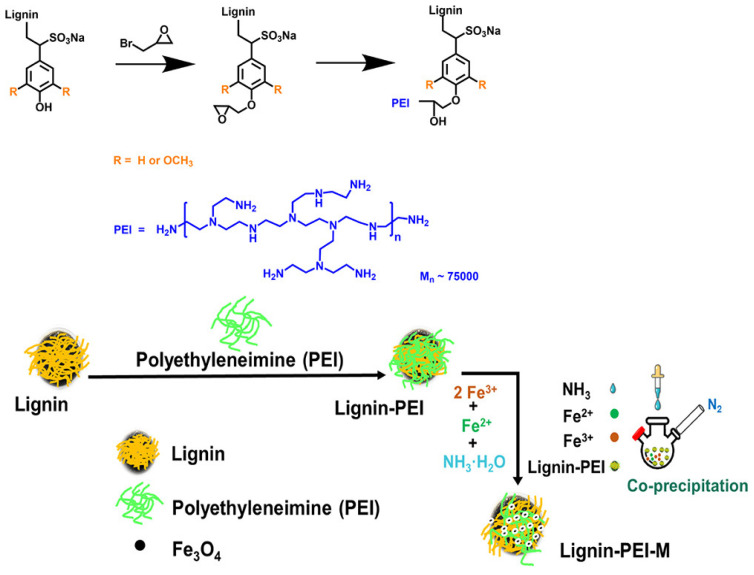
Synthesis process for a lignin-based magnetic nanocomposite biosorbent from Ref. [126]. Copyright 2021, The American Chemical Society.

By exploiting amine-functionalized lignin as a slow-release fertilizer for nitrogen, phosphorus, potassium, micronutrients, and bioactive molecules, many new materials have been produced, such as coatings, hydrogels, capsules, tablets, and nanoparticles [124,125,126,127,128,129,130,131,132]. For example, Li et al. first grafted poly(ethyleneimine) onto epoxidized lignin and then coprecipitated it with iron to synthesize amine-functionalized magnetic lignin nanocomposite bio-adsorbents. The maximum adsorption capacity of this nano-biosorbent for phosphorus was approximately 46.4 mg⋅g^−1^. The seedling study confirmed that the nano-biosorbent adsorbed with phosphorus has better root development and biomass accumulation as a recycled fertilizer (Figure 6) [126]. In the same year, another research team synthesized magnetic lignin-based nanoparticles by loading Fe_3_O_4_ nanoparticles and Fe^3+^ chelating on the aminated lignin [127], which was modified by the Mannich reaction with triethylenetetramine and formaldehyde. The nanoparticles adsorbed phosphorus from wastewater to obtain phosphorus-saturated nanoparticles, which were then used as renewable slow-release compound fertilizers. Phosphorus-saturated nanoparticles slowly increased the cumulative release of iron and phosphorus, reaching 67.2% and 69.1% of the soil after 30 days, respectively. After nutrients were released, magnetic particles could be separated from water or soil by magnets with a high recovery rate and could be regenerated again for phosphate recovery. Li et al. [128] prepared a double-layer slow-release fertilizer coating material, which used polyvinyl alcohol and methylcellulose as the inner coating materials and attapulgite doped into polyacrylic acid (super absorbent polymer) and polyacrylamide grafted lignin as the outer coating materials. Single-layer and double-coated fertilizers released urea molecules (>30 days), with cumulative release rates of urea molecules of 92.4% and 85.1%, respectively. Liu et al. [129] used a redox free radical-induced polymerization technique to synthesize a black liquor-based hydrogel with acrylic acid, *N*,*N’*-methylene bisacrylamide, and ammonium persulfate. The hydrogel was used as a water-retention material and a slow-release fertilizer. The hydrogel had an obvious positive effect on plant growth in the mimicking planting experiment. Zhu et al. [130] prepared aminated rice straw/oxidized sodium alginate/iron (III) (ARS/OSA/Fe^3+^) as a slow-release fertilizer. ARS/OSA/Fe^3+^ was small circular flakes with a diameter of 2–2.5 mm and a thickness of 1 mm, as well as with an iron content of 9.9 wt.%. It showed good slow-release performance in soil. The iron (III) release rates after 1 day and 30 days were 2.2% and 64.0%, respectively.

### 4.3. Application in Bio-Based Adhesive

Adhesives are used in diverse applications everywhere in both daily life and industry, including packaging, construction, manufacturing, and so on [133,134,135,136,137,138,139,140,141]. Petroleum-based adhesives have environmental pollution and pose risks to human health [142,143]. Therefore, the use of renewable, environmentally friendly, and clean resources to replace petroleum-based resources to prepare bio-based adhesives has attracted widespread attention. Nitrogenous functionalized lignin materials are also applied in bio-based adhesives. Recently, Saman Ghahri et al. [144] prepared acetone soluble and insoluble hardwood Kraft lignin, which was aminated with different amines (ethylenediamine, diethylenetriamine, or monoethanolamine) and cross-linked with different aldehydes (formaldehyde or glyoxal) to obtain bio-based wood adhesives. Diethylenetriamine and ethylenediamine aminated lignin contain high nitrogen contents. Diethylenetriamine aminated acetone-insoluble hardwood Kraft lignin cross-linked using both aldehydes (glyoxal and formaldehyde) exhibited excellent adhesion strength with plywood, meeting the requirements of Korean standards. Chen et al. [145] synthesized lignin-aminated waterborne polyurethane adhesives with different branched structures by introducing aminated sodium lignosulfonate as a hydrophilic chain extender. Adding 0.75% by weight of aminated sodium lignosulfonate in an appropriate way prepared polyurethane dispersions with excellent stability and high-solid content. The adhesive properties of lignin-aminated waterborne polyurethane adhesives were improved, essentially due to the entanglement of long branched chains generated by the extension of sodium lignosulfonate amine chains with other polyurethane chains, as well as the physical cross-linked network formed by the interactions between functional groups. Lignin-based wood adhesives were prepared using aminated lignin–Cu nanoparticles by Zhu et al. [146]. The lignin–Cu nanoparticles were prepared in water via a sedimentation method. Amination modification was realized by grafting amine-terminated hyperbranched polyamide onto nanoparticles. The prepared adhesive demonstrated remarkable bonding strength, debonding work, water resistance, and mildew resistance. In addition, Santos et al. [147] reported that low concentrations of unmodified lignin can be incorporated into polyurethane formulations, improving adhesive mechanical properties. The chemical modification of lignin improved its reactivity, solubility, and practical adhesion. Modified lignin allowed for the development of polyurethane adhesives with a wide range of properties, i.e., from highly brittle wood adhesives to largely flexible thermoplastic polyurethanes.

### 4.4. Applications in Coatings

**Figure 7:**
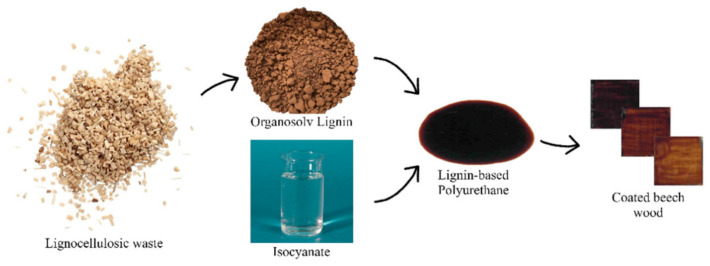
The preparation of the organosolv lignin-based PU coating for the hydrophobic function on the beech wood surface from Ref. [149]. Copyright 2022, MDPI.

The lignin-based polyurethanes used as coatings exhibit excellent antibacterial, hydrophobic, and mechanical properties and UV resistance. It may be desirable to remark on the excellent mechanical properties of lignin independently of the environmental moisture [148] and how it positively impacts the development of smart lignin-based polyurethane coatings with enhanced insulation response [60]. Bergamasco et al. [149] synthesized three different formulations of bio-based polyurethane coatings by changing the mass ratio between organosolv lignin and commercial isocyanates. The contact angle decreased with the increase in lignin content and was always lower than that of standard PU coating. Hence, the prepared lignin-based PU coating made the surface of a raw wood significantly more hydrophobic (Figure 7). Wang et al. [150] prepared lignin-based polyurethane sublayer coatings by coupling lignin-corrosion inhibitors with diisocyanate. The coating not only has certain anti-corrosion properties but also has good anti-corrosion properties. Moreover, when the coating is destroyed, a thin barrier layer is generated to reduce the corrosion rate and improve the service life. Wang et al. [151] investigated the protective effect of a polyaniline/lignin (PL)-reinforced WPU coating on cement-based materials. It was found that the introduction of PL, the hydrogen bond between lignin composite material and the isocyanate group, enhanced the cross-linking interaction of lignin polyurethane coating, resulting in improved mechanical properties and an improved water contact angle of the lignin polyurethane coatings. The impermeability of mortar with the lignin polyurethane coatings improved. This green and effective waterborne coating shows great potential in civil engineering applications.

### 4.5. Application in the Biomedical Field

**Figure 8:**
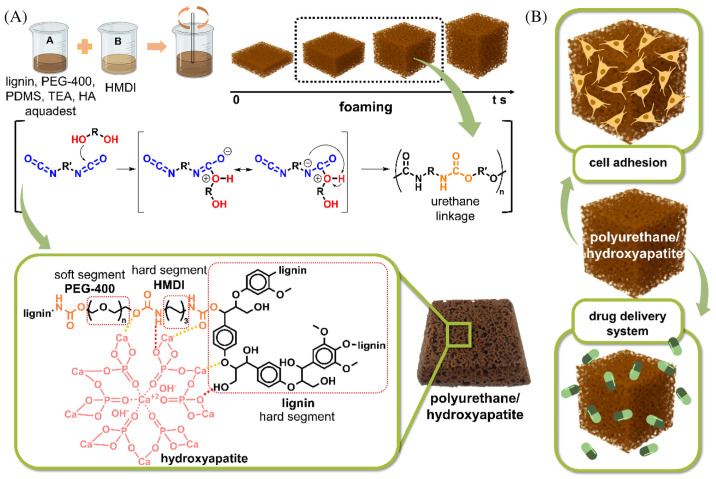
(**A**) The synthesis process and plausible chemical reaction for the formation of lignin-based polyurethane/hydroxyapatite composites. (**B**) The potential application of the composite in drug delivery systems and cell scaffolds from Ref. [157]. Copyright 2024, WILEY.

With the rapid development of lignin chemistry and materials science, the properties of lignin have been discovered to be antioxidant and antibacterial, anti-ultraviolet, anti-fluorescence, biocompatibility, and so on. A lot of lignin-based nanomaterials, carbon quantum dots, polyurethanes, and other lignin-based materials have also experienced rapid development in biomedical applications [29,152,153,154,155,156], and nitrogenous lignin-based materials are one of the promising directions. For example, Nugroho et al. [157] synthesized lignin-based polyurethane/hydroxyapatite (PU/HA) composite materials as a cell scaffold, while partially replacing polyethylene glycol 400 with lignin of oil palm empty fruit bunches and as a drug delivery system (Figure 8). PU/HA is a non-cytotoxic composite, and its cellular affinity has been improved compared to PU. The loading and release studies of quercetin have shown efficient drug loading and controlled release kinetics, with HA content and polymer relaxation exerting notable influence. Pahlevanneshan et al. [158] synthesized porous nanocomposite polyurethane foam, which contains nano-lignin (NL) and is coated with natural antimicrobial propolis for wound dressing. PU foam was synthesized with polyethylene glycol, glycerol, nano-lignin, 1, 6-diisocyanato-hexane, and water as a blowing agent. The obtained foam was immersed in ethanolic extract of propolis (EEP). The foam showed excellent biocompatibility with L929 fibroblasts, and PU-NL/EEP had the highest cell viability and cell attachment. In vivo, the Wistar rat full-thickness skin wound model was used to carry out a wound healing experiment, which confirmed that PU-NL/EEP had higher wound healing efficacy than other foams. Yi et al. [159] prepared pH-responsive charge reversal nanospheres with softwood Kraft lignin as a curcumin drug carrier through two-step modification. Firstly, softwood Kraft lignin was grafted with imidazole groups by the Mannich reaction and then synthesized with acetylated histidine-modified lignin through acetylation reaction. The drug loading degree was 22.3%, and the encapsulation efficiency was 70.8%. The in vitro release behavior of curcumin exhibits an excellent pH-dependent release property, releasing 76.8% at pH 5.7 and only 12.92% within 120 h at pH 7.4. In addition, Pang et al. [160] used diethylenetriamine (DETA) as a nitrogen source and prepared nitrogen-doped CQDs (N-CQDs) with excellent photoluminescence (PL) properties from alkali lignin (C-source) via a facile, green, and large-scale solvothermal approach. N-CQDs in ethanol (N-CQDs-ET) displayed excellent sensitivity for the detection of Fe^3+^ and Co^2+^, with good linear correlation ranges from 0.27 to 250 μM and 0.45–500 μM, respectively. Moreover, they exhibited excellent biocompatibility and multicolored cell imaging ability, demonstrating enormous potential applications in biosensors.

## 5. Conclusions

As discussed above, significant progress has been made in preparations and applications of the lignin-based nitrogenous functional materials in the past few years. The research and development of lignin-based nitrogenous functional materials have broad prospects and are expected to bring innovation and breakthroughs in many fields. Lignin can be catalyzed to produce nitrogenous bio-based chemicals, such as benzylamine and aniline. Lignin-based nitrogenous functional materials can be used to treat pollutants in sewage, especially heavy metal ions and organic dyes, and can also be used to prepare slow-release fertilizers and improve the utilization rate of fertilizers. They can also be used as drug carriers to control drug release due to their biocompatibility and degradability. Nitrogen-containing lignin-based carbon materials can be used to prepare energy storage devices such as supercapacitors and lithium-ion batteries, and they can also be used as catalysts or catalyst carriers for various chemical reactions, such as alkylation, polymerization, and so on. However, despite tremendous progress, there are still some unresolved scientific issues and techno-economic challenges in the process of lignin industrialization. For example, in the process of lignin amine functionalization, the intricate structure of lignin (depending on the plant species, growth environment, growth duration, and extracted methods), the complex processes, the harsh reaction conditions, expensive catalysts, toxic solvents, and other problems hinder the industrial development of lignin, and new simple and economical alternative technologies are needed. These are also issues that the functional modification of lignin needs to face.

In addition, because of the complex structure of lignin, it is feasible to increase the nitrogen content or a certain nitrogen-containing functional group in lignin compared to obtaining a single amination product from lignin. This will lay the foundation for the subsequent development of NIPU and nitrogen-doping carbon materials. For the synthesis of lignin-based NIPU, the cross-linking of cyclic carbonates and lignin-based polyamines is the main synthesis method. However, there is little research on the production of lignin-derived amines in the upstream process of NIPU precursors. The technical barriers of lignin-derived amines (low stability, low reactivity, and low amino content) need to be continuously overcome. Therefore, so far, current research is still far from achieving the ideal NIPU using fully renewable molecules.

In conclusion, future research will focus on improving nitrogen doping efficiency, accurately controlling nitrogen species types, increasing material properties, achieving large-scale production, developing environmentally friendly preparation methods, and exploring new application areas. These studies not only contribute to the high-value utilization of lignin but also provide new ways for the sustainable utilization of biomass resources. It is believed that lignin-based nitrogenous chemicals and functional materials will make significant contributions in many fields in the future.

## Figures and Tables

**Table 1:** Types of lignin.

Lignin Type		Solubility	Characteristics
Alkali lignin	Kraft lignin	Alkali, organic solvents	Lower sulfur content, high molecular weight (M_w)_
	Soda lignin	Alkali	Sulfur-free, lower M_w_, high p-hydroxyl, carboxyl groups
Lignosulfonates		Water, some organic solvents	High sulfur content, high M_w_, polydispersity index
Organosolv lignin		Most organic solvents	High purity, sulfur-free, lower M_w_, hydrophobic
Enzymatic lignin		Minority organic solvents	Sulfur-free, high impurity

**Table 2:** Preparation methods and performances of amine-modified lignin in wastewater treatment.

Types of Lignin	Preparation Methods	Performance	**Ref.**
Kraft lignin	Copolymerization with acrylamide in a UV-induced manner and then modified with formaldehyde and diethylenetriamine	99.3% removal rate for kaolin >99.6% decolorization rates for Acid Black 1, Direct Red 80, and Reactive Red 2	[100]
Ethyl acetate -soluble hydrothermal lignin	Copolymerization with (2-methacryloyloxyethyl) trimethyl ammonium chloride under low-power ultraviolet light and in the presence of a photo-initiator	99.6% removal rate for *E. coli*	[101]
Lignosulfonate	Copolymerization with [2-(methacryloyloxy) ethyl] trimethyl ammonium chloride under short-wavelength ultraviolet light	>95% removal rate for Acid Black 1 99.2% removal rate for kaolin 97.5% removal rate for *E. coli*	[102]
Organosolv lignin	Copolymerization with diethylenetriamine and formaldehyde	>90% decolorization rate for DB 1 dye 502.7 mg⋅g^−1^ adsorption capacity for DB 1 dye	[103]
Kraft lignin	Oxidization with H_2_O_2_, followed by copolymerization with triethylenetetramine and formaldehyde.	99.1% removal rate for malachite green	[104]
Alkaline lignin	Copolymerization with aminopropyl triethoxy silane and formaldehyde, and then cross-linking to the surface of magnetic Fe_3_O_4_ nanoparticles	234.27 mg⋅g^−1^ adsorption capacity for methylene blue	[105]
Enzymatic lignin	Magnetically recyclable functionalized (EL-PEI@Fe_3_O_4_-Mg) enzymatic lignin composite was prepared by Mannish reaction and hydrolysis–precipitation	92% removal rate for Congo red after 5 cycles	[106]
Alkaline lignin	Amine-functionalized lignin microspheres (AL-PEI/GMS) were prepared by reverse-phase suspension polymerization	99% removal rate for U(VI) in high-salinity solutions >95% removal rate for U(VI) via a fixed-bed column system	[107]
Alkaline lignin	Copolymerization with an amine and formaldehyde	>99% removal rates for Ag(I), Hg (II), Cd (II), Pb (II), Cu (II) and Zn (II) 99.7% and 99.8% removal rates for *E. coli* and *S. aureus* by Ag@LBNT, respectively	[108]
Organosolv lignin	Amine-functionalized magnetic lignin nanoparticles were prepared using cyanuric chloride as a chemoselective cross-linker	>85% removal rate for Pb (II) after 5 cycles. 111.23 mg⋅g^−1^	[109]
Organosolv lignin	Hybrid nanoparticles were prepared using epichlorohydrin as a cross-linker between amine-functionalized magnetic nanoparticles and carboxymethylated lignin	150.33 and 70.69 mg⋅g^−1^ adsorption capacities for Pb (II) and Cu (II), respectively	[110]
Industrial lignin	Polyethyleneimine-functionalized chitosan-lignin (PEI-CS-L) composite sponge was synthesized by cross-linking and lyophilization	>83.5% removal rate for Hg (II) within 1 min	[111]
Kraft lignin	Copolymerization with poly (ethylene imine) grafting agent and epoxy chloropropane cross-linker	74.84, 54.20, 53.12, and 49.42 mg⋅g^−1^ adsorption capacities for Cd (II), Cr (VI), As (V), and Ni (II), respectively	[112]
Alkaline lignin	Copolymerization with triethylenetetramine and formaldehyde, and then Fe (III) was chelated onto the aminated lignin	>90% removal rate for phosphate	[113]

## Data Availability

No new data were created or analyzed in this study.
